# Genetic diversity goals and targets have improved, but remain insufficient for clear implementation of the post-2020 global biodiversity framework

**DOI:** 10.1007/s10592-022-01492-0

**Published:** 2023-01-16

**Authors:** Sean Hoban, Michael W. Bruford, Jessica M. da Silva, W. Chris Funk, Richard Frankham, Michael J. Gill, Catherine E. Grueber, Myriam Heuertz, Margaret E. Hunter, Francine Kershaw, Robert C. Lacy, Caroline Lees, Margarida Lopes-Fernandes, Anna J. MacDonald, Alicia Mastretta-Yanes, Philip J. K. McGowan, Mariah H. Meek, Joachim Mergeay, Katie L. Millette, Cinnamon S. Mittan-Moreau, Laetitia M. Navarro, David O’Brien, Rob Ogden, Gernot Segelbacher, Ivan Paz-Vinas, Cristiano Vernesi, Linda Laikre

**Affiliations:** 1 The Morton Arboretum, Center for Tree Science, Lisle, USA; 2 The University of Chicago, Chicago, USA; 3 School of Biosciences, Cardiff University, Cardiff, UK; 4 South African National Biodiversity Institute, Pretoria, South Africa; 5 Department of Biology, Colorado State University, Fort Collins, USA; 6 School of Natural Sciences, Macquarie University, Sydney, NSW Australia; 7 NatureServe, Biodiversity Indicators Program, Arlington, USA; 8 School of Life and Environmental Sciences, Faculty of Science, The University of Sydney, Sydney, Australia; 9 INRAE, University Bordeaux, Biogeco, Cestas France; 10 U.S. Geological Survey, Wetland and Aquatic Research Center, Gainesville, USA; 11 Oceans Division, Natural Resources Defense Council, NewYork, USA; 12 Chicago Zoological Society, Species Conservation Toolkit Initiative, Brookfield, USA; 13 Conservation Planning Specialist Group, IUCN SSC, Auckland, New Zealand; 14 Centre for Research in Anthropology (CRIA), NOVA FCSH, Lisbon, Portugal; 15 Australian Antarctic Division, Department of Climate Change, Energy, the Environment and Water, Kingston, Australia; 16 Comisión Nacional para el Conocimiento y Uso de la Biodiversidad (CONABIO), Mexico City, Mexico; 17 Consejo Nacional de Ciencia Y Tecnología (CONACYT), Mexico City, Mexico; 18 School of Natural and Environmental Sciences, Newcastle University, Newcastle Upon Tyne, UK; 19 Department of Integrative Biology; Ecology, Evolution, and Behavior Program, Michigan State University, AgBio Research, Lansing, USA; 20 Research Institute for Nature and Forest, Geraardsbergen, Belgium; 21 Group on Earth Observations Biodiversity Observation Network (GEO BON), McGill University, Montreal, Canada; 22 Kellogg Biological Station; Ecology and Evolutionary Biology, Michigan State University, Lansing, USA; 23 Estación Biológica de Doñana (EBD-CSIC), Seville, Spain; 24 NatureScot, Inverness, Scotland, UK; 25 Royal (Dick) School of Veterinary Studies and the Roslin Institute, University of Edinburgh, EH25 9RG, Midlothian, United Kingdom; 26 Wildlife Ecology and Management, University Freiburg, Freiburg, Germany; 27 Forest Ecology Unit, Fondazione Edmund Mach, Trento, Italy; 28 Department of Zoology, Stockholm University, Stockholm, Sweden; 29 Centre for Ecological Genomics and Wildlife Conservation, University of Johannesburg, Johannesburg, South Africa

**Keywords:** Adaptive capacity, Gene flow, Global conservation policy, Effective population size, Indicators

## Abstract

Genetic diversity among and within populations of all species is necessary for people and nature to survive and thrive in a changing world. Over the past three years, commitments for conserving genetic diversity have become more ambitious and specific under the Convention on Biological Diversity’s (CBD) draft post-2020 global biodiversity framework (GBF). This Perspective article comments on how goals and targets of the GBF have evolved, the improvements that are still needed, lessons learned from this process, and connections between goals and targets and the actions and reporting that will be needed to maintain, protect, manage and monitor genetic diversity. It is possible and necessary that the GBF strives to maintain genetic diversity within and among populations of all species, to restore genetic connectivity, and to develop national genetic conservation strategies, and to report on these using proposed, feasible indicators.

## Background

*Destruction of habitat, overharvest, and other societal activities are leading to widespread and precipitous declines in genetic diversity* (Des Roches et al. 2021; Hoban et al. 2021a)—which is the foundation of species’ ability to adapt and a key component of ecosystem function and resilience. DNA-based studies have documented high genetic diversity losses over the past 50 to 100 years—especially in island species (28% loss), and harvested fish species (14% loss) (Pinsky and Palumbi 2014; Leigh et al. 2019). Expected genetic diversity loss due to decreased population sizes and lost habitat are also severe. A recently established mathematical relationship between population loss and genetic diversity loss from several plant and animal species suggests that genetic diversity within IUCN Threatened species has declined, on average, 9 to 33% over the past few decades (Exposito-Alonso et al. 2022). Meanwhile, population genetic theory combined with the Living Planet Index forecasts that, unless interventions are taken to stop and reverse species’ population declines, populations may ultimately lose an average of 19 to 66% of their genetic (allelic) diversity (Hoban et al. 2021a).

Genetic diversity loss has consequences for species, including determining reproduction and survival rates of individual organisms, vulnerability to climate change, and risk of species’ extinctions (Des Roches et al. 2021; Hoban et al. 2021a). Loss of genetic diversity also disrupts nutrient cycling in forests and streams (and other ecosystem services), seasonal timing of fish and bird migration, and temperature tolerance in amphibians (LeRoy et al. 2007; Schweitzer et al. 2011; Caprioli et al. 2012; Manhard et al. 2017; Bodensteiner et al. 2021). On the other hand, successful conservation of genetic diversity can increase resilience of forests and other ecosystem service providers to pests and disease, and the potential to restore coral reefs and seagrasses (Hughes and Stachowicz 2004; Budde et al. 2016; Baums et al. 2019).

One principal global mechanism for conserving biodiversity is the Convention on Biological Diversity (CBD), an international treaty among nearly all countries (plus the European Union; hereafter signatories to the Convention are referred to as “Parties”) to conserve, sustainably use, and share benefits arising from biodiversity. The CBD had multiple frameworks since it came into force in 1993, including the Strategic Plan from 2002 to 2010, with four strategic goals for biodiversity and underlying targets for each (CBD 2002, 2004); the Strategic Plan for Biodiversity 2011–2020, known as the Aichi Biodiversity Targets (CBD 2010); and ongoing preparations for a post-2020 global biodiversity framework (GBF, https://www.cbd.int/conferences/post2020, CBD 2022a). The post-2020 GBF is expected to have four high level goals for 2050 related to the state of nature resulting from conservation, nature’s contributions to people and its sustainable use, shared benefits arising from biodiversity, and means of implementation and resource mobilization; and 22 action targets on changes in human society and activities needed by 2030 to achieve the goals. The GBF is being negotiated and must be agreed upon by all Parties, and therefore reflects scientific input, political negotiation, perceived feasibility, and compromise.

To navigate developments in the post-2020 GBF text with respect to genetic diversity, over the past three years, we provide a scientific synthesis of its past, present and possible future status. We aim to identify specific and science-informed improvements that could strengthen the GBF. We describe: the progression of wording around genetic diversity in GBF goals and targets, up to October 2022; highlight issues to be resolved in the final GBF draft, with suggestions for resolving them; share lessons from participating in this process; and reiterate connections between GBF wording and indicators to measure progress under the monitoring framework of the GBF.

Box 1 Reflections on pre-2020 commitments The original 1992 CBD convention text (https://www.cbd.int/convention/text/) outlines many commitments—in situ and ex situ conservation, sustainable use, protected areas, research, public education—needed for the “conservation of biological diversity… [which] includes diversity within species, between species and of ecosystems.” The 2002–2010 commitments and the Strategic Plan for 2011–2020 contained a high-level goal and a target for genetic diversity (see Table 1), but had major issues such as vague wording and a focus on agricultural and other socioeconomically and culturally ‘valuable’ species. This led to national monitoring and reporting on genetic diversity that was primarily concerned with crops and livestock, crop wild relatives, and harvested trees, and on ex situ activities like seed banks and agricultural breeding programs, while neglecting most wild species (Hoban et al. 2020b,2021c). As one exception to this trend, Scotland produced a sub-national scorecard assessing genetic diversity in wild species as part of their progress toward Aichi Target 13 (Hollingsworth et al. 2020). Terms that were not well defined included “maintain genetic diversity,” “minimize genetic erosion,” and “safeguard genetic diversity.” *We suggest that defined terminology is vital in setting targets to ensure consistent, transparent and effective translation to national actions and measurement.*

**Table 1:** Wording from prior CBD commitments (first two rows) and proposed draft text from three years of CBD negotiations for genetic diversity in the post-2020 global biodiversity framework goals and targets (row three onwards)

Document	Goal	Target	Comments
2002–2010 commitments (CBD 2002)	Promote the conservation of genetic diversity	Genetic diversity of crops, livestock, and harvested species of trees, fish, and wildlife and other valuable species conserved, and associated indigenous and local knowledge maintained	This commitment was only clarified in COP7, in (CBD 2004)
Strategic Plan 2011–2020 (CBD 2010)	Strategic Goal C “Improve the status of biodiversity by safeguarding ecosystems, species and genetic diversity”	Target 13: By 2020, the genetic diversity of cultivated plants and farmed and domesticated animals and of wild relatives, including other socio-economically as well as culturally valuable species, is maintained, and strategies have been developed and implemented for minimizing genetic erosion and safeguarding their genetic diversity	2011–2020 was also the UN Decade on Biodiversity
Zero draft GBF, January 2020 (CBD 2020b)	B. 2030 and 2050 Goals (c) “Genetic diversity is maintained or enhanced on average by 2030, and for [90%] of species by 2050”	No target mentioning genetic diversity, only mention in targets is benefits of genetic resources	
Updated Zero draft e.g. 0.5 draft, August 2020 (CBD 2020c)	Goal A “The area, connectivity and integrity of natural ecosystems increased by at least [X%], supporting healthy and resilient populations of all species while reducing the number of species that are threatened by [X%] and maintaining genetic diversity”	Same as above	This Goal A formulation was based on proposals and comments after the zero draft (CBD 2020d)
First draft GBF, July 2021 (CBD 2021b)	Goal A “…genetic diversity of wild and domesticated species is safeguarded, with at least 90 per cent of genetic diversity within all species maintained.”	Target 4 “Ensure active management actions to enable the recovery and conservation of species and the genetic diversity of wild and domesticated species, including through ex situ conservation, and effectively manage human-wildlife interactions to avoid or reduce human-wildlife conflict.”	Goal A contains phrases for each of ecosystems, species and genetic diversity, but for brevity we list only the genetic phrase
Report of OEWG 3, March 2022 (CBD 2022b)	Goal A “…[All genetically distinct populations and] [[[a] A]t least [90][95][X] per cent of] genetic diversity among and within [all] [known] [populations of] [wild and domesticated] species is [maintained] [safeguarded, maintaining their adaptive potential].]” Milestone A.3 “Genetic diversity of wild and domesticated species is safeguarded, with an increase in the proportion of species that have at least 90 per cent of their genetic diversity maintained”	“…and the genetic diversity of [[native] wild and domesticated] [cultivated] [all] [native] [and domesticated] species [populations], [to maintain their adaptive potential] including through in situ [conservation, supported by] [and] ex situ conservation [and restoration of genetically depleted populations]…”	As above, the full wording of Target 4 and Goal A are not presented; only the genetic diversity element Note, participants of OEWG 3 part 2 agreed to simplify the structure of the post-2020 GBF, particularly removing the 2030 milestones for tracking progress. Proposals were made to integrate the contents of the milestones into the goals and targets
Report of OEWG 4, June 2022 (CBD 2022c)	Goal A, Option 1 “…[The genetic diversity and adaptive potential of [all] [known] [wild and domesticated] species is safeguarded and [all genetically distinct populations are] maintained [by 2030, at least [95] per cent of genetic diversity among and within populations of [native] [wild and domesticated] species is maintained by 2050].]” Goal A, Option 2 “… maintaining genetic diversity of populations and their adaptive potential [numerical values to be added]	“…[and] [to] [maintain and restore] the [genetic diversity] [within and between populations] of [all species] [[all] [native] wild and domesticated species]] [[to] [and] maintain their adaptive potential] including through in situ and ex situ conservation,…]”	During OEWG 4, two options for Goal A were presented (see left). Both have three elements (ecosystems, species, and genetic diversity). Option 2 was shorter, and less specific, but was not discussed and was recommended for further discussion at COP 15
Informal Group recommendations, October 2022 (CBD 2022d)	*Identical to OEWG4 text*	“…to maintain and restore the genetic diversity [within and between populations] of [all] [native] wild and domesticated species [to maintain their adaptive potential], including through in situ and ex situ conservation,…”	The informal group recommended keeping the words “maintain and restore” but to consider whether “adaptive potential” was duplicative

Each document was released by the CBD Secretariat, typically after negotiation sessions among Parties. Brackets denote portions of text that have not reached consensus and are still under negotiation by Parties. Each new text supersedes previous text and thus the last row of the table is the most recent text to be used for ongoing negotiation

Target 13 also called for developing national strategies for genetic conservation, though guidance on developing or reporting such strategies were lacking. We are not aware of the existence of many such national strategies.* Still, frameworks for assessing wild species’ genetic diversity in situ and ex situ show that it is feasible and beneficial to publish such national or subnational reports* (Hollingsworth et al. 2020; Hoban et al. 2020; O’Brien et al. 2022).

Another issue was the lack of indicators for tracking and reporting on genetic diversity for populations of wild species (Hoban et al. 2020). Consequently, most Parties did not report for their progress towards Aichi Target 13 (https://www.cbd.int/aichi-targets/target/13, CBD 2020a), while a scientific assessment of mid-term progress on the Strategic Plan (Tittensor et al. 2014) only quantified the status of threatened domestic breeds. The final assessment of Aichi Target 13 indicates that the genetic diversity of cultivated plants, farmed and domesticated animals, and wild relatives is continuing to erode and that the target has not been achieved (CBD 2022a). As such, Díaz et al. (2020) called for more ambitious objectives for genetic diversity in the post-2020 GBF, and the CBD and others have acknowledged gaps in genetic diversity indicators for wild species (CBD 2016, 2021a; OECD 2019).

## Assessment of progress on integrating genetic diversity into the CBD framework

Largely due to the COVID-19 pandemic, finalizing the post-2020 GBF has taken two years longer than originally anticipated. Nonetheless, delays did give Parties extended time to meet, discuss, and improve the GBF. Delays also emphasized a need for clear and ambitious GBF target wording with relevant indicators to catalyze immediate and aligned action for genetic diversity.

The January 2020 GBF “zero draft” (CBD 2020b) Goal A only vaguely referenced genetic diversity (Table 1). It seemed to suggest that many species could continue to lose genetic diversity (“on average”), and that by 2050, it was acceptable for up to 10% of species to be losing genetic diversity. The August 2020 “updated zero draft” (CBD 2020c) reverted to Goal wording of simply “maintaining” genetic diversity, yet neither draft had a target for genetic diversity. Overall, the GBF in late 2020 risked serious regression around genetic conservation compared to the 2002 and 2011 commitments.

The first draft of the post-2020 GBF (CBD 2021b) provided a quantitative goal for the first time, Goal A, on maintaining genetic diversity, though only “…genetic diversity of wild and domesticated species is safeguarded, with at least 90% of genetic diversity within all species” relative to a baseline of 2020. This percentage had been suggested by Díaz et al (2020). To non-geneticists, 90% may sound satisfactory, but Frankham (2022) demonstrated that maintaining 97% at 2050 and thus 90% after 100 years will lead to catastrophic increases in inbreeding, and “a 54% loss of total fitness in naturally outbreeding vertebrate populations and 30% loss in outbreeding plants,” sending many species into an irreversible ‘extinction vortex’ (Blomqvist et al. 2010). Additionally, the wording around genetic diversity in the 2030 Milestones (which were later removed) remained confusing (“increase in the proportion of species that have at least 90 per cent of their genetic diversity maintained”). In contrast, genetic diversity appeared for the first time in Target 4—recognizing (a) that genetic diversity conservation requires management actions and that (b) populations of already imperiled species are experiencing genetic threats (inbreeding, lost populations, lack of connection to other populations, etc.). Target 4 was a new Target focusing on active management interventions (e.g. captive breeding, translocations, supplemental feeding) needed for species and populations that would not recover on their own after threats or pressures were removed (such threats being the focus of Targets 1–3 and 5–8) (Bolam et al 2022).

The report of the Open-ended Working Group of the Post-2020 GBF in March 2022 (CBD 2022b) contained suggestions from Parties to the CBD, with many suggestions in brackets for later negotiations. The new bracketed text of Goal A included possible improvements in clarity and specificity regarding genetic diversity, including several suggested previously (Hoban et al. 2020b, 2021b; Laikre et al. 2021) (the culmination of extensive outreach effort, see 2: Lessons Learned). The percentage of genetic diversity to maintain was increased to 95%, and the text was modified to emphasize genetic diversity ‘among and within populations’ and recognize ‘adaptive potential’. Maintenance of genetically distinct populations was also mentioned. Lastly, Target 4 added more specificity, highlighting “restoration of genetically depleted populations” and various wording such as “all species”, “ex situ and in situ conservation,” “species’ populations”, etc. Although other documents were released later in 2022 (see Table 1), no substantial changes occurred after OEWG 3.

Overall, from January 2020 to March 2022, progress was remarkable for genetic conservation concepts in the GBF. Genetic diversity in Goal A evolved from a simple mention, to a quantitative commitment, to higher ambition in the quantitative commitment and more specificity, to clearer connections to recently developed indicators. Genetic diversity in Target 4 evolved from no mention to a simple mention, to more specificity and ambition.

Vital elements of the current text are worth highlighting. Specifically, genetic diversity *among populations* (e.g. genetic differentiation) is needed to maintain unique adaptations to different local environments, while genetic diversity *within populations* is needed to allow each population to avoid inbreeding and quickly respond to changing conditions (Turner et al. 2008; Flood and Hancock 2017; Bitter et al. 2019). This helps the overall species, and each population, to survive. Both are needed, and maintaining one does not necessarily result in maintaining the other (Forester et al 2022)*.* Retaining this concept will strengthen the post-2020 GBF, preferably as ‘maintain all genetically distinct populations and genetic diversity within populations’ (detailed in next section).

### Issues that remain

Improvements in the post-2020 GBF draft are laudable, and we believe reflect an increasing acceptance among CBD policy makers of the importance of genetic diversity and of the demonstrated feasibility of measuring it with simple indicators. However, the current wording misses some elements, and so refining the text is critical to retain key principles and ensure robust monitoring and reporting, while avoiding perverse incentives and potential loopholes.

Goal A currently includes maintenance of genetic diversity “within all species” or “within wild and domesticated species.” Either phrase is suitable, as both emphasize that genetic diversity within all species matters (note: the word “within” is critical to retain in the text).

The ambition remains insufficient at 95%, as highlighted by Frankham (Frankham 2022), who showed that no genetic diversity (specifically, heterozygosity, see Table 2) can be lost. Specifically, he showed that even maintaining 97% of present genetic diversity by 2030 or 2050 will lead to a drastic increase in inbreeding and decline in individual fitness and thus population and species viability. An additional issue with the 95% figure is that genetic diversity can be assessed with numerous metrics (allelic richness, heterozygosity, adaptive genetic variance) and each declines at different rates. Heterozygosity is the most common metric, but allelic richness responds sooner and declines much faster. Furthermore, few species globally have any population genetic data (DNA data from individuals across geographic space), and perhaps only a few dozen species, in a handful of countries, have regular temporal DNA assessments (Torres-Florez et al 2018; Posledovich et al. 2021), Therefore, Goal A must connect to an indicator that does not rely on DNA assessments directly but rather uses a measurable value, namely effective population size (Ne).

**Table 2:** Proposed definitions of technical terms

Term	Definition
Heterozygosity	A measure of genetic diversity that ranges from zero to one; higher values provide a buffer for populations during environmental change, and are correlated with higher fitness and survival
Allelic richness	The number of alleles—genetic variants—that exist. To simplify slightly, the more alleles, the more “options” a population or species has—the more various possible future environments it can adapt to. It is analogous to maintaining species diversity in an ecosystem
Genetic drift	The loss of genetic diversity to random chance—a sampling effect at each generation. Genetic drift is higher in smaller populations. Analogous to the loss of species in small fragments. Rare alleles are usually lost first
Effective population size (Ne)	A metric that measures the rate of loss of genetic diversity. Ne > 500 (or Ne > 1000, see Frankham 2014, 2022) is an approximate threshold value of this metric, below which genetic diversity is rapidly lost (see Fig. 2 in Willi et al. 2021)—resulting in populations that do not maintain adaptive potential. Ne is often ~ 1/10th the census size, thus Ne = 500 corresponds to census size of approximately 5000
Genetic diversity	Genome-wide diversity existing in populations (also known as “standing genetic variation”). Standing genetic variation is a major contributor to adaptive potential
Adaptive potential	The ability of populations to evolve in response to environmental change, or the extent to which they can evolve. Adaptation occurs by changes in frequency of alleles that determine traits. Adaptive potential is a consequence of a large pool of genetic diversity and the size of the population. Typically, Ne=500 has been considered a minimum threshold to maintain adaptive potential
Safeguarded	To protect or make safe. In the context of biodiversity, to take actions to protect, for the long term, including in situ protected areas, ex situ gene banks, and other activities. The actions are designed to minimize harm
Maintained	To keep at the current state with respect to diversity level; prevent any decline or loss or diversity
Population	A geographically, genetically, ecologically, and/or behaviorally coherent and distinct group of individuals of a species

Therefore, a more scientifically justified Goal would be “maintain at least 99% of within population genetic diversity and maintain all distinct populations,” or “maintain sufficient genetic diversity for adaptive potential within populations and maintain all distinct populations.” For most populations, ‘maintain sufficient genetic diversity for adaptive potential’ implies near zero loss of current genetic diversity (or when needed, restored genetic diversity through active management) which can be reported on using indicators for effective population size of 500 within each population to mitigate loss from genetic drift (see Implementation and Reporting below). No loss is consistent with CBD’s Mission (“To take urgent action across society… to put biodiversity on a path to recovery by 2030 for the benefit of planet and people” and Vision (“living in harmony with nature”), especially as many species already suffered high genetic diversity loss (DiBattista 2008; Leigh et al. 2019; Exposito-Alonso et al. 2022). Ne ≥ 500 (or, as Frankham (2022) suggests, Ne > 1000) is important for preventing future losses for all populations within species regardless of past losses—though we acknowledge it does not address the extent of losses over previous decades and centuries. (Note that the CBD GBF has tentatively agreed that 2010-2020 will be the baseline for such percentage measures).

Finally, it is important to ensure the goal and target text are clear and not contradictory, to allow for effective implementation and measurement (see Table 2 for some definitions). The terms “safeguarded” and “maintained” may cause confusion, unless defined. Similarly, “genetic diversity” and “adaptive potential” have both been recommended previously (Hoban et al. 2020; Díaz et al. 2020). The two are not duplicative or fully interchangeable. Retaining the wording “adaptive potential” also emphasizes the need for future adaptation of populations to climate change, disease, and other challenges——a critical message for society and policy makers. See Table 3 for possible wording.

**Table 3:** Summary of proposed resolved text for Goal A, Target 4, and targets relating to “genetic connectivity” and “safeguarding genetic diversity”

Element	Proposed possibilities for final wording
Goal A	**All genetically distinct populations,** and at least 99% of **[all] genetic diversity within populations, of all species, are maintained by 2030** and genetic connectivity is restored by 2050
Goal A	**Genetic diversity and adaptive potential within populations of all [wild and domestic] species is safeguarded, and all genetically distinct populations are maintained by 2030, and at least 99% of genetic diversity within populations is maintained by 2050**
Goal A	The proportion of populations large enough **to maintain genetic diversity and adaptive potential** (Ne > 500), of all species, has increased by at least 25% by 2030 and 75% by 2050, and **all genetically distinct populations are maintained**
Target 4	**And to maintain and restore the genetic diversity and adaptive potential within and among populations of species,** and *strategies* for conserving *genetic diversity are developed and initiated***…**
Target 4	**And to maintain,** manage, protect, **and restore the genetic diversity and adaptive potential within and among populations of species,** and *strategies* for conserving *genetic diversity are developed and initiated***…**
Target 1 and 12	“**Ecological connectivity**” change to “**ecological** and genetic **connectivity**”
Target 3	“**Ecologically representative**” protected areas; change to “**ecologically** and genetically **representative**”
Target 5 and 9	“**Sustainable harvest**” change to “demographically and genetically **sustainable harvest**”

Bold shows wording from OEWG4 and italics is text in Aichi Target 13 but not in the OEWG4 document (see Table 1). We also note that achieving Target 8 on resilience under climate change and Target 10 on agriculture/ aquaculture/ forestry will require maintaining genetic diversity, though we do not suggest wording changes there. Multiple lines are given for Goal A and Target 4 to provide options for policy makers

### Missing wording

Current wording also misses several important elements of genetic conservation. The first two of these are particularly vital.

Remarkably, although the 2011–2020 Strategic Plan mandated that genetic conservation strategies be ‘developed and implemented’, this has not been included in the post-2020 GBF yet. Adding phrasing such as ‘with genetic conservation strategies in place, and implemented’ is recommended. A component indicator for reporting such conservation genetic strategies would be valuable, and easily measurable (a simple yes or no).

Gene flow (via genetic exchange, also known as genetic connectivity among populations) is also not mentioned as yet. In many situations, e.g., recent population fragmentation, maintaining genetic diversity will require appropriate levels of gene flow (which may necessitate restoring habitat connectivity or implementing periodic translocations). Therefore, it is recommended to add gene flow explicitly to the current Goal A: “ensure appropriate genetic connectivity/ gene flow” (Frankham 2022*);* or targets on connectivity could explicitly add "genetic’ (see Table 3).

Hoban and colleagues (Hoban et al. 2020) previously advocated that in addition to "maintain", genetic diversity must be protected (e.g. through policy or legislation), monitored (with indicators and/or DNA based research), and managed (e.g. active intervention). It is essential that monitoring is required for reporting on indicators, and management is required for Target 4. Explicit inclusion of these words and the word "‘protected" may contribute to assurance of maintaining genetic diversity.

Maintenance of genetic diversity is generally understood to be no loss of alleles or decrease in heterozygosity (or similar). However, it is sometimes understood as also preventing hybridization, maintenance of allele frequencies, and sustainable levels of offspring production, as mentioned by Hollingsworth and colleagues (Hollingsworth et al. 2020). These issues are not mentioned in current wording. The CBD Glossary would greatly benefit from clearly defining ‘maintain’ and include these examples.

### Implementation and reporting

Indicators exist to monitor and report on genetic diversity “among and within populations” i.e. “maintain genetic diversity within populations” and “no loss of distinct populations” as well as on monitoring and active management using DNA based studies. Data are available, indicators are SMART (Specific, Measurable, Achievable, Realistic, and Time bound), and calculations are feasible (Hoban et al. 2020, 2022). The “Ne indicator” is currently included in the GBF monitoring framework as a headline indicator (A.5, CBD 2022e, previously A.0.4 CBD 2021c) for Goal A. Also, the “distinct populations” indicator (A.8.1) has been proposed as a component indicator (CBD 2021c, d)- but is more suitable as a headline indicator. No genetic diversity headline indicator is listed under Target 4 (CBD 2021c, d); both A.5/A.0.4 and A.8.1 would be appropriate for Target 4 and possibly Targets 1 and 3.

A valuable component indicator on “safeguarding”—assuming this term means “protection”—is available for measuring in situ protected area coverage and ex situ coverage in seed and gene banks, using geographic area as a genetic diversity proxy (currently a.51, CBD 2021d); this could serve Goal A, Target 4, or Target 3, see Table 3 (Khoury et al. 2019, 2020). The “genetic scorecard” indicator (currently a.48) is also valuable for summarizing and showcasing genetic status of high-profile species in a manner that is accessible to the public and policy makers; it is also feasible and requires only moderate resources. It is not as quantitative as the “Ne” and “populations lost” indicators, but it is a holistic measure that could feature these indicators, and also relate to Target 5 (Hollingsworth et al. 2020). This indicator could also highlight formerly common populations whose Ne have declined precipitously but remain above Ne = 500. Further, indicators on threatened breeds and the size of seed/ gene banks (currently a.52 and a.53) are still important for tracking genetic diversity for food security and culture (https://www.post-2020indicators.org/), though they may be better suited as indicators for Targets 7 and 10. Lastly, we propose a complementary indicator: the number of populations or species being monitored with DNA-based methods (Hoban et al. 2020) because accumulating genetic data improves management of genetic diversity. All of these indicators are feasible for reporting on circa 2025, and again in 2030.

Beyond a clearly worded, ambitious and quantifiable goal and target for genetic diversity, it will be critical that GBF commitments are consistently translated at the national scale to ensure effective and measurable action. This will require guidance for Parties on National Biodiversity Strategies and Action Plans and sustained technical support for national conservation strategies and deployment of indicators to effectively track progress (Xu et al. 2021). Definitions of genetic diversity terminology in the CBD Glossary (CBD 2022f) for the post-2020 GBF are essential for accurate scientific measurements and progress. We propose the inclusion of definitions for “genetically distinct populations,” “genetic diversity within populations”, “maintain”, “genetic connectivity”, “adaptive capacity”, etc. (see also Table 2).

BOX 2. Lessons learned Our involvement with the post-2020 GBF led to several lessons about conservation policy, some of which have been highlighted previously (Hoban et al. 2013, 2020; Taylor et al. 2017; Holderegger et al. 2019; Taft et al. 2020; Kershaw et al. 2022). These are:

- It is vital to build relationships with decision makers- persons present in the rooms where policy is drafted and negotiated. For example, it is necessary to correspond frequently with the national representatives attending CBD meetings (https://www.cbd.int/information/nfp.shtml). Decision makers helped us evaluate the feasibility of proposals, understand perspectives of other decision makers, and present proposed wording.
- Global and two-way engagement is required. It is necessary to connect with countries spanning a diversity of economic resources, uses/ reliance on biodiversity, etc., on every continent. Dialogue, listening, and two-way knowledge sharing is also needed to provide voice to issues of national capacity, to ensure that goals, targets and indicators meet the needs and capabilities of all nations.
- Interpretation and outreach are necessary for translating scientific findings, using easy to understand language, graphics, and clear point-by-point actions needed.
- Continual engagement is required—a marathon commitment and an ability to rapidly act with very high levels of work at certain periods.
- A large team with diverse skills, background and connections is helpful. For instance, forming the Coalition for Conservation Genetics (Kershaw et al. 2022) allowed wide dissemination of policy briefs and messages, leveraging the reputation of globally respected organizations (e.g. IUCN), assistance with multi-lingual translation of documents, collaborations with NGOs, and more. Involving non-academic researchers and policy makers/framework drafters with specific academic training, also helped bridge the science-policy divide.
- Virtual interactions helped collaboration, discussion, knowledge sharing, and constructive critique. Despite technical difficulties, virtual platforms were more inclusive and allowed us to meet people we otherwise could not have, and participate with higher frequency than in person meetings allow. One regret is that although we did gather suggestions through dialogue, we collected a limited amount of systematic or quantitative feedback through surveys and polls (mentimeter, zoom, etc.).
- A diversity of engagement modes is critical: webinars, mass emails to policy makers, direct and frequent personalized emails, numerous digital meetings, journal articles, navigating bureaucracies for institutional approval, and frequent submission of document comments—often with deadlines of days to several weeks. We created three policy briefs, 13 journal articles, four Statements sent to 500 + recipients, a SBSTTA (Subsidiary Body on Scientific Technical and Technological Advice) information document, 12 + webinars (e.g. https://www.youtube.com/watch?v=Oku8eTqH_hE), dozens of email chains to various CBD stakeholders, a side event for COP 15 which was co-sponsored by 50 + institutions and NGOs, **an information booth at COP 15,** and ad hoc responses to many inquiries several multi-page comment submissions to CBD, and ad hoc responses to many inquiries.
- Currently a small fraction of scientists are involved in international biodiversity policy discussions (directly or indirectly); increasing this engagement requires both greater efforts by scientists to understand and join in policy discussions and more frequent and accessible opportunities for scientists to enter the policy realm (Laikre 2010). Bridge organizations and programmes such as (International Union for the Conservation of Nature)IUCN, G-BiKE (Genomic Biodiversity Knowledge for Resilient Ecosystems, EU COST Action), GEO BON (Group on Earth Observation Biodiversity Observation Network, geobon.org) and the SCB (Society for Conservation Biology) provide such opportunities.
- Progress depends on intensive and steadfast commitment from several ‘champions’ in research and policy, far outside their normal job obligations, to create outputs, bring people and groups together, and constantly track many moving parts.

## Conclusion

The post-2020 GBF currently includes clearer language and more measurable commitments to genetic diversity conservation, due to clearer scientific consensus and very active participation by conservation geneticists through numerous policy channels and negotiations, though there is scope for improvement in the final negotiations. The role of scientists in the CBD process needs strengthening, via greater involvement of scientists and more invitations for scientists, such as for indicator evaluation and testing (e.g. the Ad Hoc Technical Expert Group). Communication between scientific groups, the CBD Secretariat, Parties, the IUCN, IPBES (Intergovernmental Panel on Biodiversity and Ecosystem Services), GEO BON, and other stakeholders during implementation of the GBF is vital for capacity development and shared learning, which will take significant time and effort. We close by emphasizing that although “Ne > 500”, “maintain populations” and other feasible indicators can be implemented at national scales, Parties are urged to remember the spirit of the goal: little to no genetic diversity loss so that populations, species and nature retain adaptive potential.
